# Was sind anonymisierte Daten? Möglichkeiten der datenschutzkonformen Bereitstellung von Krebsregisterdaten für die Forschung

**DOI:** 10.1007/s00103-026-04305-8

**Published:** 2026-09-23

**Authors:** Joachim Hübner, Jana Johne, Maria Heil, Benjamin Barnes

**Affiliations:** 1 Klinische Landesauswertungsstelle Niedersachsen (KLast), Industriestr. 9, 26121 Oldenburg, Deutschland; 2 Vertrauensstelle des Bayerischen Krebsregisters, Bayerisches Landesamt für Gesundheit und Lebensmittelsicherheit (LGL), Nürnberg, Deutschland; 3 NOVACOS Rechtsanwälte Heil∙Hübner∙Natz∙Oeben∙Stallberg Partnerschaft mbB, Düsseldorf, Deutschland; 4 Zentrum für Krebsregisterdaten (ZfKD), Robert Koch-Institut, Nordufer 20, 13353 Berlin, Deutschland

**Keywords:** Forschungsdaten, Datenschutz, DSGVO, Anonymisierung, Krebsregister, Research data, Data protection, GDPR, Anonymization, Cancer registries

## Abstract

Im Gesundheitssystem werden täglich große Mengen patientenbezogener Daten erhoben. Voraussetzung für die Nutzung solcher Daten zu Forschungszwecken ist in der Regel, dass eine Einwilligung der betroffenen Personen vorliegt oder dass die Daten in anonymisierter Form bereitgestellt werden. Sowohl aufseiten der Forschenden als auch bei den Datenhaltern besteht häufig Unsicherheit darüber, was Anonymisierung bedeutet und wie sie erreicht werden kann. Genügt die Entfernung unmittelbar identifizierender Angaben wie Name und Anschrift? Wenn nein, mit welchem Zusatzwissen, das eine Identifikation ermöglichen könnte, muss gerechnet werden? Und kann ein Datensatz erst dann als anonymisiert gelten, wenn er bedenkenlos der Allgemeinheit zugänglich gemacht werden könnte?

Dieser Diskussionsbeitrag beleuchtet am Beispiel von Krebsregisterdaten das Spannungsfeld zwischen Forschungsbedürfnissen und den Datenschutzinteressen der betroffenen Personen. Ausgehend von der Definition personenbezogener Daten in Art. 4 Nr. 1 Datenschutz-Grundverordnung (DSGVO) und der aktuellen Rechtsprechung des Europäischen Gerichtshofs (EuGH) wird dargelegt, dass Anonymität keine ausschließlich den Daten inhärente Eigenschaft ist, sondern sie hängt vom spezifischen Reidentifikationsrisiko ab, das auch durch den Kontext der Datenbereitstellung beeinflusst wird. Der Beitrag zeigt, wie sich die Wahrscheinlichkeit der Reidentifikation sowohl durch die Aufbereitung der Daten als auch durch rechtliche Beschränkungen der Datennutzung minimieren lässt. Wenn das verbleibende Risiko bei lebensnaher Betrachtung – mit den Worten des EuGH – „de facto vernachlässigbar“ ist, sind die Voraussetzungen für eine Datenbereitstellung in anonymisierter Form erfüllt.

## Abstract (en)

Every day, large amounts of patient-related data are collected in the healthcare system. The prerequisite for using such data for research purposes is usually that the consent of the persons concerned has been obtained or that the data are provided in anonymized form. Both researchers and data holders are often uncertain about what anonymization means and how it can be achieved. Is it sufficient to remove directly identifying information such as name and address? If not, what additional information that could enable identification must be considered? And can a dataset only be considered anonymized if it can be made available to the general public without risk?

This article highlights the tension between the needs of research and the data protection interests of the individuals concerned, using the example of cancer registry data. Based on the definition of personal data in Art. 4 No. 1 of the General Data Protection Regulation (GDPR) and current case law of the European Court of Justice (ECJ), it is argued that anonymity is not a property solely inherent to the data. Rather, it depends on the specific risk of re-identification, which is also influenced by the context in which the data are to be provided. The article highlights ways in which the probability of re-identifying a data subject can be minimized, both through data pre-processing and through legal and contractual restrictions on data use. If the remaining risk is judged to be—in the words of the ECJ—“de facto negligible,” the pre-requisites for providing data in anonymized form have been met.

## Einleitung

In der medizinischen und der Public-Health-Forschung benötigte Daten enthalten in der Regel Angaben zum Gesundheitszustand von Menschen. Solche Informationen sind in hohem Maße persönlichkeitsrelevant [1] und bergen ein gesteigertes Diskriminierungsrisiko [2]. Gesundheitsbezogene Informationen könnten etwa bei beruflichen Auswahlverfahren, bei Kreditvergaben oder beim Abschluss von Versicherungsverträgen zum Nachteil der betroffenen Personen verwendet werden. Das Recht des Einzelnen, grundsätzlich selbst über die Preisgabe und Verwendung seiner personenbezogenen Daten zu bestimmen (informationelles Selbstbestimmungsrecht aus Art. 2 Abs. 1 i. V. mit Art. 1 Abs. 1 Grundgesetz – GG), hat daher im Bereich der Gesundheit eine besondere Relevanz. Für das ärztliche Behandlungsverhältnis war die Schweigepflicht bereits im hippokratischen Eid festgelegt, hat Eingang in die Genfer Deklaration des Weltärztebundes und die Musterberufsordnung für Ärztinnen und Ärzte (§ 9 MBO‑Ä 1997) gefunden und ist strafrechtlich sanktioniert (§ 203 Abs. 1 Strafgesetzbuch – StGB).

Im Rahmen klinischer Studien wird dem Selbstbestimmungsrecht Rechnung getragen, indem die Teilnehmenden in die beabsichtigte Verwendung ihrer Daten nach entsprechender Aufklärung einwilligen. Neben dem administrativen Aufwand besteht ein Nachteil einwilligungsbasierter Datensätze darin, dass sie wegen des unvermeidlichen Selbstselektionsbias kein unverzerrtes Bild der Krankheitslast und Versorgungsrealität vermitteln können. Es kann nicht davon ausgegangen werden, dass Fälle, für die eine Einwilligung vorliegt, repräsentativ für die Zielpopulation sind [3]. Viele Forschungsfragen lassen sich daher valide nur mit bereits vorhandenen bevölkerungsbezogenen Daten beantworten, die für andere als Forschungszwecke oder ohne Bezug zu einer konkreten Forschungsfrage erhoben worden sind. Beispiele sind Abrechnungsdaten der gesetzlichen Krankenversicherung, Daten der organisierten Krebsfrüherkennungsprogramme und Daten der klinischen und epidemiologischen Krebsregister der Länder. Zum Schutz des informationellen Selbstbestimmungsrechts der betroffenen Personen sehen die einschlägigen Gesetze zum Teil unterschiedliche Verfahren der Zugänglichmachung zu Forschungszwecken vor. Pseudonymisierte Abrechnungsdaten, die weder Namen noch sonstige, unmittelbar identifizierende Merkmale enthalten (siehe unten), werden gemäß § 303 e Abs. 4 Sozialgesetzbuch Fünftes Buch (SGB V) für die Verarbeitung unter Kontrolle des Forschungsdatenzentrums bereitgestellt. Analysen erfolgen ausschließlich in einer sicheren Verarbeitungsumgebung auf einem virtuellen, über das Internet erreichbaren Desktop. Auch das Gesundheitsdatennutzungsgesetz (GDNG) sieht vor, pseudonymisierte Einzeldaten nur in einer sicheren Verarbeitungsumgebung zugänglich zu machen (§ 4 Abs. 5 GDNG^1^). Ziel ist, die Verarbeitung durch die Antragstellenden auf das für den jeweiligen Nutzungszweck erforderliche Maß zu beschränken und insbesondere ein Kopieren der Daten zu verhindern. Die – aus Sicht der betroffenen Personen eingriffsintensivere – Übermittlung von Abrechnungsdaten setzt hingegen voraus, dass diese zuvor anonymisiert wurden (§ 303e Abs. 3 SGB V, entsprechend für die Weitergabe durch datenverarbeitende Gesundheitseinrichtungen § 6 Abs. 3 GDNG). Auch Daten der organisierten Krebsfrüherkennungsprogramme und Daten des Zentrums für Krebsregisterdaten nach dem Bundeskrebsregisterdatengesetz (BKRG) werden in anonymisierter Form übermittelt (§ 25a Abs. 5 SGB V bzw. § 8 Abs. 1 BKRG). Die Landeskrebsregistergesetze, die als Spezialgesetze den Bestimmungen der Landesdatenschutzgesetze vorgehen, sehen ebenso wie die EU-Verordnung (EU) 2025/327 über den europäischen Gesundheitsdatenraum (EHDS) die Datenbereitstellung in anonymisierter Form zumeist als Regelfall vor. Erwägungsgrund 26 der europäischen Datenschutz-Grundverordnung (DSGVO) stellt fest, dass die Verordnung auf anonyme Daten nicht anwendbar ist. Es wird deutlich, dass die Frage nach der Anonymität von Daten in der medizinischen und Public-Health-Forschung von großer Relevanz ist.

Die Praxis der Rechtsanwendung zeigt allerdings, dass über die inhaltliche Bedeutung von Anonymität Unsicherheit besteht. Während Datenschutzbehörden oft strenge Anforderungen stellen, neigen Forschende einem weniger restriktiven Verständnis zu. Im Folgenden gehen die Autoren unter Berücksichtigung der aktuellen Rechtsprechung des Europäischen Gerichtshofs (EuGH) der Frage nach, was Anonymisierung rechtlich bedeutet und wie sich Datenanonymität praktisch umsetzen lässt. Als Praxisbeispiel dienen die Daten der Landeskrebsregister, die ähnlich wie die Abrechnungsdaten der Krankenkassen einen großen, für die Forschung nutzbaren Bestand an Gesundheitsdaten darstellen.

## Vorgaben der DSGVO

Maßstab für die Begriffsbestimmung ist die DSGVO, die als unmittelbar geltendes Recht bundes- und landesrechtlichen Regelungen vorgeht. Abweichende Bestimmungen auf der Ebene der Mitgliedstaaten, die zu einer Unterschreitung des gemeinschaftsrechtlichen Schutzniveaus führen, sind nur zulässig, soweit sie sich im Rahmen von Öffnungsklauseln bewegen. Eine ausdrückliche Definition anonymer oder anonymisierter Daten findet sich in der DSGVO nicht. Wie sich aus Erwägungsgrund 26 ergibt, ist Anonymisierung der Vorgang, bei dem Daten ihre Eigenschaft verlieren, „personenbezogen“ zu sein [4]. Personenbezogene Daten sind nach Art. 4 Nr. 1 DSGVO „alle Informationen, die sich auf eine identifizierte oder identifizierbare natürliche Person … beziehen“. Die erste Alternative bereitet keine Schwierigkeiten. Hier ergibt sich die Identität der Person unmittelbar aus der Information selbst. Das ist der Fall, wenn die Daten ausreichend Merkmale enthalten, die typischerweise zur eindeutigen Bezeichnung einer Person verwendet werden, z. B. Name, Anschrift und Geburtsdatum. Vielfach wird angenommen, dass die Entfernung solcher unmittelbar identifizierenden Informationen aus einem Datensatz genügt, um die Daten zu anonymisieren. Das ist häufig aber nicht der Fall. Denn nach der zweiten Alternative (Identifizierbarkeit) ist der Personenbezug bereits gegeben, wenn sich die Identität der betroffenen Person durch Hinzuziehung zusätzlichen Wissens ergibt.

Im Sonderfall einer Pseudonymisierung von Daten existiert solches Wissen in Form von Zuordnungsfunktionen, mittels derer identifizierende Merkmale betroffener Personen (z. B. Name, Geburtsdatum) durch einen Code (z. B. eine Buchstaben- oder Zahlenkombination) ersetzt werden. Die Trennung von pseudonymisierten Daten und Zuordnungsregel durch technisch-organisatorische Maßnahmen reduziert das Risiko, dass ein Personenbezug hergestellt werden kann, schließt dies jedoch zumindest in der Sphäre des Verantwortlichen nicht aus [5]. Die Bedeutung der Pseudonymisierung für die Forschung liegt darin, dass sie die Zusammenführung von Einzeldatensätzen aus unterschiedlichen Quellen oder aus unterschiedlichen Entstehungszeiten ohne Namensnennung ermöglicht.

Unabhängig von einer Pseudonymisierung kann sich die Identifizierbarkeit aus Zusatzwissen ergeben, das Dritte über betroffene Personen haben und das zugleich in den Daten enthalten ist. Die DSGVO nennt beispielhaft Standortdaten, physische, genetische oder psychische Merkmale. Treten bestimmte Merkmalsausprägungen oder Kombinationen davon in den Daten nur einmal auf, kann jeder, der eine Person mit entsprechenden Eigenschaften kennt und weiß, dass sie in den Daten enthalten ist, diese Person herausfiltern („aussondern“ im Sinne von Erwägungsgrund 26 der DSGVO). Seltene Ausprägungen, z. B. ein Lebensalter von 105 Jahren, bergen ein besonders hohes Reidentifikationsrisiko. Merkmale, die unerwünschte Reidentifikationen ermöglichen, werden Quasiidentifikatoren genannt; Personen, die unerwünschte Reidentifikationsversuche unternehmen, werden als Angreifer bezeichnet.

## Reidentifikationsrisiken in Krebsregisterdaten

Krebsregisterdaten enthalten neben Angaben zur Person detaillierte Information zur Diagnose, zur durchgeführten Behandlung und zum Krankheitsverlauf. Ein Angreifer, der wenig, aber spezifisches Zusatzwissen über einen Fall hat, z. B. das Alter der oder des Betroffenen, den Tag einer Operation und die Diagnose betreffend, hat gute Chancen, den Fall auch ohne unmittelbar identifizierende Informationen durch einfaches Filtern im Datensatz zu finden. Auch Wissen um die Art und das Jahr einer früheren Tumorerkrankung, die Lokalisation einer Fernmetastase oder die Art einer angewandten Therapie (z. B. Chemotherapie) kann zur Identifikation beitragen. Kritisch, weil spezifisch und oft bekannt, ist auch das Sterbedatum. Zwar gilt die DSGVO nicht für Daten Verstorbener. Nationale Gesetzgeber sind jedoch nicht daran gehindert, Daten Verstorbener als Teil des postmortalen Persönlichkeitsrechts zu schützen (Erwägungsgrund 27 DSGVO). Entsprechende Regelungen finden sich in verschiedenen Landeskrebsregistergesetzen.

## Datenbezogene Anonymisierungstechniken

Vor diesem Hintergrund stellt sich die Frage, wie Krebsregisterdaten in der Praxis anonymisiert werden können. Die Antwort liegt auf 2 Ebenen:

Die erste Ebene betrifft Umfang und Inhalt der bereitzustellenden Daten. Die einzuschließenden Fälle, die Auswahl der Items und die darzustellenden Ausprägungen sind auf das für die Forschungsfrage notwendige Maß zu beschränken. Besondere Aufmerksamkeit gilt Attributen mit engem Personenbezug. Wenn der beantragte Datensatz seltene Ausprägungskombinationen dieser Merkmale enthält, beispielsweise Melanom bei Kindern oder Brustkrebs bei Männern, ist kritisch zu prüfen, ob solche Daten für das Auswertungsvorhaben zwingend erforderlich sind. Fälle, deren Geschlecht weder weiblich noch männlich ist, haben aufgrund ihrer Seltenheit ein besonders hohes Identifikationsrisiko. Zum Schutz der Persönlichkeitsrechte der Betroffenen ist es in der Regel erforderlich, solche Fälle bei der Datenzusammenstellung auszuschließen. Weiter kann durch eine Vergröberung feingranularer Daten eine Zuordnung der Datensätze zu einer konkreten betroffenen Person erschwert werden. An die Stelle des Geburtsdatums und des Tags der Diagnose können oft das Diagnosejahr und das Alter bei Diagnose in Jahren treten. Seltene Altersstufen können oft ohne Verlust der Aussagekraft durch breitere Altersklassen ersetzt werden. Entsprechend können an die Stelle des Diagnosejahres mehrjährige Perioden treten. Solche Vergröberungen schließen taggenaue Analysen von Überlebenszeiten oder Behandlungsverläufen (z. B. Time-to-treatment-Analysen) nicht aus. Zu diesem Zweck bietet es sich an, die exakten Datumsangaben der interessierenden Ereignisse durch die Zahl der Tage seit der Diagnose zu ersetzen. Werden Regionalmerkmale wie PLZ, Kreis oder Gemeinde beantragt, sollte die räumliche Auflösung nicht höher sein, als es die Fragestellung erfordert. Zudem ist zu prüfen, ob der Analysezweck auch ohne Mitlieferung des Regionalmerkmals erreicht werden kann. Dient es dazu, einem Einzeldatensatz einen regionalen Indikator (z. B. Siedlungsstruktur oder sozioökonomische Deprivation) zuzuordnen, so kann diese Zuordnung bereits bei der Erzeugung des Lieferdatensatzes im Register erfolgen. Hierfür stellt der Antragsteller eine Liste mit Regionen und dem jeweils zuzuordnenden Indikator zur Verfügung. Für einen systematischen Überblick über gebräuchliche Anonymisierungstechniken wird auf die wissenschaftliche Spezialliteratur [3, 4] und Praxisleitfäden [6–8] verwiesen.

Abstrakt formuliert zielen die Techniken überwiegend darauf ab, die Ähnlichkeit der Einzeldatensätze in Bezug auf Quasiidentifikatoren zu erhöhen. Inwieweit das gelingt, ist als k‑Anonymität statistisch beschreibbar. Ein Gesamtdatensatz ist k‑anonym, wenn für jede Person mindestens k – 1 weitere Personen dieselbe Kombination von Ausprägungen der Quasiidentifikatoren aufweisen. Die insoweit übereinstimmenden Einzeldatensätze bilden eine Äquivalenzklasse. Bestimmte Ausprägungen sensibler Merkmale lassen sich einzelnen Personen einer Äquivalenzklasse nur noch mit der Wahrscheinlichkeit zuzuordnen, die der relativen Häufigkeit dieser Ausprägung in der Äquivalenzklasse entspricht. Wenn für alle oder einen großen Teil der Personen dieselbe sensible Information (z. B. „hat Erkrankung x“) enthalten ist, sind trotz gegebener k‑Anonymität die Persönlichkeitsrechte der betroffenen Personen gefährdet (sog. Homogenitätsangriff bei mangelnder Diversität). Konzepte, die Unzulänglichkeiten der k‑Anonymität kompensieren, sind l‑Diversität und t‑Closeness [6].

Neben solchen Anonymisierungstechniken, die die Granularität von Daten reduzieren und vergröbern, gibt es sog. perturbative Methoden. Sie verzerren Daten, indem sie Werte des Ausgangsdatensatzes durch unzutreffende Werte ersetzen. Dabei wird versucht, die statistischen Beziehungen des Datensatzes weitgehend zu erhalten. Einen Extremfall stellen synthetische Daten dar: künstliche Datensätze, die auf der Basis modellierter statistischer Beziehungen des Ausgangsdatensatzes erzeugt werden [9]. Aus wissenschaftlicher Perspektive können verzerrte, insbesondere synthetische Daten problematisch sein, da sie den Informationsgehalt des Ausgangsdatensatzes nicht bloß reduzieren, sondern im ungünstigen Fall unrichtige Evidenz erzeugen oder verstärken können [10]. Sie eignen sich damit eher für hypothesengenerierende als für konfirmatorische Forschung.

Die Manipulation von Daten zum Zweck der Anonymisierung stellt nach wohl herrschender, aber nicht unbestrittener [11] Meinung eine Datenverarbeitung dar, die einer Rechtsgrundlage bedarf [12]. Zum Teil wird dies ausdrücklich auf Situationen beschränkt, in denen der Personenbezug durch Löschung identifizierender Merkmale unwiederbringlich beseitigt wird [13]. Wird hingegen – wie es bei der Bereitstellung von Forschungsdaten durch die Krebsregister stets der Fall ist – nur eine (auszugsweise) Kopie des Datensatzes ohne Personenbezug erstellt, ist nach dieser Auffassung keine besondere Rechtsgrundlage erforderlich. Sofern eine Rechtsgrundlage für die Übermittlung anonymisierter Daten existiert, kann im Übrigen davon ausgegangen werden, dass diese den Vorgang der Anonymisierung als notwendige Voraussetzung umfasst. Dies ist in den hier betrachteten Situationen der Fall. Soweit sich aus der Anonymisierung in der Gesamtschau ein hohes Risiko für die Rechte und Freiheiten natürlicher Personen ergibt, kann die Durchführung einer Datenschutz-Folgenabschätzung nach Art. 35 DSGVO notwendig sein [6].

Für die Anwendung verschiedener Anonymisierungstechniken stehen mehrere frei verfügbare Tools zur Verfügung [14]. Selbst mit ihrer Hilfe ist das Idealziel eines vollständig anonymen Datensatzes, der jedes Reidentifikationsrisiko ausschließt und gleichzeitig ausreichend informativ für ein Forschungsprojekt mit multivariaten Analysen bleibt, in der Regel nicht erreichbar.

## Kontext der Datenbereitstellung

Folgt man besonders restriktiven Auffassungen, können Daten erst dann als anonym gelten, wenn sie ohne Gefährdung der Rechte betroffener Person veröffentlicht werden können. Soweit also geltendes Recht die Bereitstellung von Daten in anonymisierter Form fordert, wären viele Forschungsvorhaben zumindest mit diesen Daten nicht durchführbar. Diese Sichtweise verkennt, dass Datenanonymität als Rechtsbegriff auf einer zweiten Ebene die Umstände der Datenbereitstellung und -verarbeitung einbezieht. Ob ein Datensatz anonym ist oder nicht, hängt nach dem Begriff der Identifizierbarkeit davon ab, welches zusätzliche Wissen genutzt werden kann. In einem viel beachteten Urteil hat der EuGH jüngst klargestellt, dass es für dieses Kriterium auf die Perspektive des Datenempfängers ankommt [15]. Zu berücksichtigen ist allerdings nicht nur solches Wissen, das der Datenempfänger bereits hat, sondern auch solches, das er sich verschaffen kann. Eine bloß theoretische Möglichkeit genügt hier wiederum nicht. Wäre dies so, würde jedem Datenempfänger sämtliches Wissen zugerechnet, das an irgendeinem Ort der Welt verfügbar ist („gesamtes Weltwissen“). Vielmehr sind gemäß DSGVO (nur) solche Mittel zu berücksichtigen, die „nach allgemeinem Ermessen wahrscheinlich“ genutzt werden, um die betroffene Person zu identifizieren (Erwägungsgrund 26 DSGVO). Dazu gehören nach der Rechtsprechung des EuGH insbesondere Informationen, die im Internet verfügbar sind [16]. Die Feststellung, ob Daten hinreichend anonymisiert sind, erfordert danach eine lebensnahe Risikobeurteilung. Bei der Prognose sind neben den „Kosten der Identifizierung und [dem] dafür erforderlichen Zeitaufwand“ auch rechtliche Beschränkungen zu berücksichtigen, denen der Datenempfänger unterliegt [17]. Wirksame Beschränkungen reduzieren nicht nur die Gefahr, dass der Datenempfänger einen Personenbezug herstellt, sondern auch die Gefahr, dass Dritte Zugang zu den Daten erhalten. Dadurch würde sich das potenziell nutzbare Zusatzwissen erweitern. Auch wenn die Ausformung dieser Kriterien in der Rechtsprechung noch nicht abgeschlossen ist: Als Konsequenz der umfassenden Beurteilungsgrundlage ist festzuhalten, dass Anonymität keine ausschließlich den Daten inhärente Eigenschaft ist, sondern auch vom erwarteten Kontext der Datennutzung abhängt. Am risikoarmen Ende des Spektrums steht die Bereitstellung von Daten an einen oder wenige namentlich benannte Forschende, die strengen Nutzungsbedingungen unterworfen sind. Die Nutzungsbedingungen beinhalten in der Regel neben der Vorlage eines Datenschutz- und Datensicherheitskonzepts, das Maßnahmen gegen unbefugte Offenlegung und unbefugten Zugang zu den Daten festlegt, auch das Verbot der Weitergabe an Dritte, konkrete Löschungsfristen und Regeln für wissenschaftliche Publikation. Sofern keine Anhaltspunkte gegen die Zuverlässigkeit der Forschenden sprechen, wird sich ein Krebsregister darauf verlassen können, dass die Bedingungen der Datenbereitstellung von den Datenempfängern beachtet werden. Alternativ lassen sich solche Beschränkungen durch Bereitstellung von Daten in einer kontrollierten, digitalen Auswertungsumgebung realisieren. Am risikoreichen Ende des Spektrums von Datenbereitstellungen steht die Veröffentlichung in einem öffentlichen Bericht eines Registers oder als Public Use File. Aufgrund des unbegrenzten Zugangs zu den Daten ist hier das Risiko, dass eine betroffene Person identifiziert wird, viel größer. Es besteht etwa die Gefahr, dass jemand individuelles Zusatzwissen nutzt, um etwas über den Gesundheitszustand einer ihm persönlich bekannten Person, etwa des Nachbarn, herauszufinden. Zu denken ist auch an Fälle, in denen jemand aus Neugier oder journalistischem Interesse versucht, eine Person des öffentlichen Lebens in den Daten zu identifizieren. Das hierfür notwendige Zusatzwissen haben manche betroffene Personen durch transparenten Umgang mit ihrer Erkrankung selbst öffentlich gemacht. Daraus kann jedoch nicht geschlossen werden, dass weitere in den Krebsregisterdaten enthaltene Informationen (z. B. prognostisches Stadium, sexuelle Funktionsstörung als Nebenwirkung einer Therapie) ebenfalls bekannt werden sollen.

## Die Praxis der Krebsregister

Die Entwicklung standardisierter Vorgehensweisen stellt wegen der Unterschiedlichkeit der Forschungsvorhaben und der Vielschichtigkeit der Bewertungsaufgaben eine Herausforderung dar. Das Zentrum für Krebsregisterdaten (ZfKD) am Robert Koch-Institut und teilweise auch die Landeskrebsregister orientieren sich am „Five-Safes“-Modell. Das Modell erfasst daten- und kontextbezogene Faktoren in 5 Dimensionen: Safe Project, Safe People, Safe Data, Safe Settings und Safe Outputs. Zur näheren Information wird auf die Spezialliteratur verwiesen [18, 19]. Gegenstand der Entscheidung über die Bereitstellung von Krebsregisterdaten ist zumeist nicht der Antrag in seiner ursprünglichen Form. Vielmehr werden der Zuschnitt der Daten und die Bedingungen der Bereitstellung in der Regel in Abstimmung mit dem Antragsteller festgelegt, um den Datenbedarf zur Beantwortung primärer und sekundärer Forschungsfragen einerseits und Datenschutzerfordernisse andererseits bestmöglich zum Ausgleich zu bringen.

Aktuell erfolgt die Abstimmung mit dem Antragsteller noch auf Ebene der einzelnen Krebsregister. Für die Zukunft erarbeiten die Krebsregister eine gemeinsame Struktur, die einen Teil der Antragserfordernisse einheitlich klärt und als zentrale Anlaufstelle für Antragstellende dient.

## Fazit

Der Begriff der „anonymisierten Daten“ erweist sich bei näherer Betrachtung der Vorgaben der Datenschutz-Grundverordnung (DSGVO) als komplex. Für die Bereitstellung von Daten zu gesetzlich zulässigen Zwecken verlangt die DSGVO nicht, dass eine Reidentifikation einzelner Personen unter allen Umständen ausgeschlossen ist. Vielmehr muss die Wahrscheinlichkeit einer Reidentifikation im jeweiligen Kontext des Forschungsvorhabens bewertet werden. Nur wenn sich ergibt, dass das Risiko – mit den Worten des Europäischen Gerichtshofs [17] – „de facto vernachlässigbar“ ist, dürfen die Daten bereitgestellt werden. Dieses wertende Element und die Kontextabhängigkeit der Anonymität machen es für viele Forschungsvorhaben möglich, Bedürfnisse der Forschung mit den Datenschutzinteressen der Betroffenen in Einklang zu bringen.

## Data Availability

Für diesen Artikel wurden keine Datensätze erstellt oder analysiert.

## Footnotes

1 Nach aktuellem Gesetzentwurf zukünftig § 27 Abs. 5 GDNG (Stand: 28.05.2026).

## References

[1] BVerfG Beschluss v. 19. Mai 2020 – 1 BvQ 1/20.

[2] Hartung J (2024) Kommentierung zu Art. 4 Nr. 15. In: Kühling J, Buchner B (Hrsg) DS-GVO, 4. Aufl. C.H.Beck,

[3] El Emam K, Rodgers S, Malin B (2015) Anonymising and sharing individual patient data. BMJ 350:h1139 10.1136/bmj.h1139 PMC4707567 25794882

[4] Hölzel J (2018) Anonymisierungstechniken und das Datenschutzrecht. DuD 2018(8):502–509

[5] Weichert T (2022) Datenschutzrechtliche Rahmenbedingungen medizinischer Forschung. Vorgaben der EU-Datenschutz-Grundverordnung und national geltender Gesetze. Medizinisch Wissenschaftliche Verlagsgesellschaft, Berlin 10.32745/9783954667000 (Zuletzt zugegriffen: 31. Mai 2026)

[6] Stiftung Datenschutz (2022) Praxisleitfaden für die Anonymisierung personenbezogener Daten. https://stiftungdatenschutz.org/fileadmin/Redaktion/Dokumente/Anonymisierung_personenbezogener_Daten/SDS_Studie_Praxisleitfaden-Anonymisieren-Web_01.pdf. Zugegriffen: 31. Mai 2026

[7] Benschop T, Welch M. (o.D.). Statistical Disclosure Control for Microdata: A Practice Guide. https://sdcpractice.readthedocs.io/en/latest/ Zuletzt zugegriffen: 31. Mai 2026

[8] Bundesverband der Deutschen Industrie (BDI) (2020) Anonymisierung personenbezogener Daten: Ein branchenübergreifender Praxisleitfaden für Industrieunternehmen. https://bdi.eu/de/publications/anonymisierung-personenbezogener-daten. Zugegriffen: 31. Mai 2026

[9] Kokosi T, Harron K (2022) Synthetic data in medical research. BMJ Med 1(1):e000167 10.1136/bmjmed-2022-000167 PMC9951365 36936569

[10] Azizi Z, Zheng C, Mosquera L, Pilote L, El Emam K, GOING-FWD Collaborators (2021) Can synthetic data be a proxy for real clinical trial data? A validation study. BMJ Open 11(4):e043497 10.1136/bmjopen-2020-043497 PMC8055130 33863713

[11] Thüsing G, Rombey S (2021) Anonymisierung an sich ist keine rechtfertigungsbedürftige Datenverarbeitung. Eine Auslegung von Art. 4 Nr. 2 DS-GVO nach den Methoden des EuGH. ZD 2021(10):548–553

[12] BfDI (2020) Positionspapier zur Anonymisierung unter der DSGVO unter besonderer Berücksichtigung der TK-Branche (2020). https://www.bfdi.bund.de/SharedDocs/Downloads/DE/Konsultationsverfahren/1_Anonymisierung/Positionspapier-Anonymisierung.pdf. Zugegriffen: 31. Mai 2026 (Der Bundesbeauftragte für den Datenschutz und die Informationsfreiheit)

[13] Hartung J (2024) Kommentierung zu Art. 4 Nr. 2. In: Kühling J, Buchner B (Hrsg) DS-GVO, 4. Aufl. C.H.Beck,

[14] Francis P, Jurak G, Leskošek B, Otte K, Prasser F (2025) Comparison of Three Anonymization Tools for a Health Fitness Study. Sci Data 12(1):1548 10.1038/s41597-025-05823-x PMC12446462 40968117

[15] EuGH (2025) Urteil v. 04.09.2025 – C‑413/23 P, ECLI:EU:C:2025:645.

[16] EuGH (2024) Urteil v. 07.03.2024 – C‑479/22 P, ECLI:EU:C:2024:215.

[17] EuGH (2016) Urteil v. 19.10.2016 – C‑582/14, ECLI:EU:C:2016:776

[18] Desai T, Ritchie F, Welpton R (2016) Five Safes: Designing data access for research. Working Paper. 2016. University of the West of England: Economics Working Paper Series, 1601. https://www2.uwe.ac.uk/faculties/BBS/Documents/1601.pdf. Zugegriffen: 31. Mai 2026

[19] Wirth FN, Meurers T, Johns M, Prasser F (2021) Privacy-preserving data sharing infrastructures for medical research: systematization and comparison. BMC Med Inform Decis Mak 21(1):242 10.1186/s12911-021-01602-x PMC8359765 34384406

